# Intraoperative and postoperative outcomes of Harmonic Focus versus monopolar electrocautery after neoadjuvant chemotherapy in breast conservative surgery: a comparative study

**DOI:** 10.1186/s12957-021-02435-8

**Published:** 2021-11-15

**Authors:** Islam Khaled, Ihab Saad, Hany Soliman, Mohammed Faisal

**Affiliations:** 1 Department of Surgery, Faculty of Medicine, Surgical Oncology Unit, Suez Canal University, Kilo 4.5, Ring Road, Ismailia, Egypt; 2 Saudi German Hospital, Jeddah, Saudi Arabia; 3 Surgical Oncology Department, National Cancer Institute, Cairo University, Cairo, Egypt; 4 Department of Clinical Oncology, Faculty of Medicine, Cairo University, Cairo, Egypt

**Keywords:** Breast cancer, Conservative surgery, Neoadjuvant chemotherapy, Harmonic scalpel

## Abstract

**Background:**

Surgical devices are commonly used during breast conservative surgery (BCS) to provide better hemostasis. The Harmonic scalpel has recently gained momentum as an effective tool for intraoperative bleeding reduction. This comparative study was designed to determine the efficacy of Harmonic Focus in reducing postoperative complications of BCS after neoadjuvant chemotherapy (CTH) compared to the conventional method using monopolar diathermy.

**Results:**

A prospective, nonrandomized, comparative study was conducted on patients scheduled to undergo BCS with axillary dissection after neoadjuvant CTH. Patients in the Harmonic Focus group had significantly shorter operative times than the monopolar electrocautery group (101.32 ± 27.3 vs. 139.3 ± 31.9 min, respectively; *p* < 0.001). Besides, blood loss was significantly lower in the Harmonic Focus group (117.14 ± 35.6 vs. 187 ± 49.8 mL, respectively; *p* < 0.001). Postoperatively, patients in the Harmonic Focus group had a significantly lower volume of chest wall drain (*p* < 0.001) and shorter time until drain removal (*p* < 0.001). Likewise, patients in the Harmonic Focus group had a significantly lower volume of axillary drain and shorter time until drain removal than monopolar electrocautery (*p* < 0.001). The incidence of postoperative complications was comparable between both groups (*p* = 0.128).

**Conclusions:**

This study confirmed the superiority of Harmonic Focus compared to monopolar electrocautery among patients receiving neoadjuvant CTH before BCS.

## Introduction

Breast conservative surgery (BCS) has emerged as a promising technique for patients wishing to preserve their breast, with comparable survival rates to conventional mastectomy [1]. Recent literature demonstrated that BCS has increased in locally advanced breast cancer (BC) to reach up to 80% in some cohorts, especially after neoadjuvant chemotherapy. Although it was initially proposed for inoperable locally advanced BC, many centers have recently popularized neoadjuvant CTH in earlier operable cases [2]. The emergence of taxanes and other novel agents has dramatically improved the pathological response after neoadjuvant CTH and the clinical outcomes of the patients [3].

Despite being generally safe, BCS is not a complications-free procedure; the surgery can be complicated by postoperative wound infection, seroma, dehiscence, bleeding, and thromboembolic events [4]. Neoadjuvant CTH can potentially increase the risk of postoperative complications, especially infection, owing to its associated neutropenia [5]. Nonetheless, the impact of neoadjuvant CTH on the postoperative complications of BCS is still controversial. Therefore, meticulous efforts have been dedicated to reducing the risk of postoperative complications of BCS after neoadjuvant CTH by innovating new surgical devices.

Surgical devices are commonly used during BCS to provide better hemostasis of small blood vessels, thus reducing the risk of bleeding and prolonged operation [6]. Conventional electrocautery is a widely available, easy-to-use, and cheap method for blood vessel sealing during surgery; however, the tool is limited by the induction of postoperative inflammatory reactions and wide burn area, which can increase the risk of postoperative seroma; besides, excessive smoking from electrocautery may compromise the surgical field [7]. In a recent systematic review, electrocautery was associated with the highest incidence of postoperative seroma among surgical devices for BCS [8]. The Harmonic scalpel has recently gained momentum, mainly in laparoscopic surgery, as an effective tool for intraoperative bleeding reduction [9]. In patients scheduled for BC surgery, the Harmonic scalpel has been investigated to reduce postoperative seroma incidence with equivocal results [10, 11].

This comparative study was designed to determine the efficacy of Harmonic Focus in reducing postoperative complications of BCS after neoadjuvant CTH compared to the conventional method using monopolar diathermy.

## Materials and methods

Before the study’s initiation and first patient enrollment, official approval of the responsible ethics committee was obtained from Suez Canal University Hospital and Saudi German Hospital, Jeddah (institutional review board approval nos. 3128 and 124, respectively).

### Study design and population

A prospective, nonrandomized comparative study was conducted at Suez Canal University Hospital and Saudi German Hospital, Jeddah, from January 2017 to December 2019. Adults (ages >18 years) who were scheduled to undergo BCS with axillary dissection biopsy after neoadjuvant CTH were included. Cases with a history of disease recurrence and radiation therapy and those who refused to sign the informed consent were excluded. Patients were recruited consecutively throughout the study period and randomly assigned in a 1:1 ratio to undergo either monopolar electrocautery or Harmonic Focus. Eligible patients were randomized by a computer program (www. Randmizer.org), and allocation sequences were done by opaque closed envelopes.

### Sample size calculation

The sample size was calculated using G*Power version 3.1.9.2 for Windows. According to Bohm et al., patients who underwent Harmonic Focus showed less intramammary seroma than the conventional technique (5.7% vs. 20.3%; *p* = 0.042). Setting the *α*-error at 5% and the power at 80%, the sample size was calculated to be 82 patients (41 patients per group) without accounting for the dropout rate.

### Preoperative data collection and surgical techniques

Data regarding demographic characteristics, body mass index (BMI), history of chronic disease, histopathological type of tumor, hormonal status, tumor size, tumor stage, neoadjuvant CTH regimen, and radiological response were collected from all patients. With immunohistochemistry done for all patients, all patients received CTH regimens in the form of doxorubicin and cyclophosphamide [Adriamycin, cyclophosphamide (AC)] followed by paclitaxel (Taxol) for four cycles; Herceptin was added to the patients with Her2neu overexpression. The clinical and radiological assessment of the response was done every four cycles. The radiological response to neoadjuvant CTH was assessed according to the Response Evaluation Criteria in Solid Tumors (version 1.1).

**Fig. 1:**
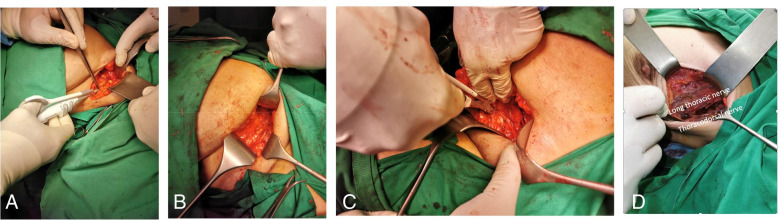
**A** Dissection of breast tissue using the Harmonic scalpel. **B** After wide local excision of *left* breast mass. **C** Dissection of axilla. **D** Postaxillary dissection with the Harmonic scalpel

All patients underwent BCS according to the institution’s standard protocols after neoadjuvant CTH. ErbeVio 300 D was utilized in the monopolar diathermy group to dissect the skin flap, with wide local excision, a safety margin, and axillary dissection, as indicated. In the Harmonic Focus group, Harmonic Focus was utilized for wide local excision along with axillary dissection. Blood and lymphatic vessels were sealed using Harmonic Focus without any attempts to use cautery or clips. In patients who underwent BCS, after identifying the nerve supply, Harmonic Focus was used to ligate veins and arterial supply of the resected segment of the breast and dissect axial lymph nodes and the surrounding blood vessels (Fig. 1). Finally, two 16-F vacuum drains were allocated in the chest wall and axilla. Intraoperative blood loss was measured by calculating the weight of used sponges, with each gram corresponding to milliliter of blood loss.

Broad-spectrum antibiotics were prescribed for 12 h after the operation. Postoperative pain was recorded until the seventh postoperative day using a Visual Analog Scale. The assessment of the surgical site for infection or necrosis was done until the patients’ discharge. Patients were discharged with drains and instructed to measure the daily amount of drain volume. The drains were removed within an average of 5 days after the operation. The patients were followed up weekly for 4 weeks to assess the development of seroma itself according to the definition of seroma, which is accumulation of fluid beneath the skin flaps.

### Study endpoints

The primary endpoint of this study was the incidence of postoperative seroma and the mean seroma cumulative volume 30 days after the surgery. The seroma was diagnosed by ultrasound or subcutaneous aspiration-proven serious fluid beneath the skin flaps to the extent that causes the patient’s discomfort within 1 month after the operation. The secondary endpoints included intraoperative blood loss, operative time, amount of chest wall and axillary drain, days till chest wall and axillary drain removal, hospital stay, postoperative pain, and incidence of postoperative complications.

### Statistical analysis

Statistical data analysis was done using Microsoft Excel 2013 (15.0.4420.1017) 32-bit software. The mean ± standard deviation (SD) and frequencies were used to summarize continuous and categorical data. The hypothesis of the association between qualitative data was tested using *χ*^2^ or Fisher’s exact test, whereas the hypothesis of the association between quantitative data was performed using the Mann–Whitney test. A *p*-value of less than 5% was used to reject the null hypothesis.

## Results

A total of 100 patients were divided into a 1:1 ratio to undergo Harmonic Focus or monopolar electrocautery. One patient in the Harmonic Focus group was lost during the follow-up period. Thus, 99 patients were included in the final dataset analysis. The mean ± SD age in the monopolar electrocautery and the Harmonic Focus groups was 49.5 ± 11.3 and 48.1 ± 11.1 years, respectively (*p* = 0.53). There were no statistically significant differences between the studied groups in terms of sex (*p* = 0.51), BMI (*p* = 0.51), smoking (*p* = 0.78), family history of BC (*p* = 0.96), history of other chronic diseases, histological types (*p* = 0.32), receptor status, tumor size, tumor-node-metastasis (TNM) stage (*p* = 0.15), CTH regimens, and radiological response (*p* = 0.72; Table 1).

**Table 1:** Preoperative data of the studied groups

Variables		HF group (***n*** = 49)	CE group (***n*** = 50)	***p***
**Age (years), mean ± SD**		49.51 ± 11.3	48.1 ± 11.1	0.53
**Females,** ***n*** **(%)**		48 (98)	48 (96)	0.51
**BMI (kg/m**^**2**^**), mean ± SD**		30.65 ± 5.4	31 ± 6.5	0.78
**Smoking,** ***n*** **(%)**		7 (14.3)	7 (14)	0.96
**Family history,** ***n*** **(%)**		2 (4.1)	2 (4)	0.98
**Comorbidities,** ***n*** **(%)**	DM	13 (26.5)	12 (14)	0.77
	HTN	13 (26.5)	12 (14)	0.77
	Others	15 (30.6)	21 (42)	0.24
**Histological type,** ***n*** **(%)**	Invasive duct carcinoma	41 (83.7)	42 (84)	0.32
	DCIS	4 (8.2)	7 (14)	
	Invasive lobular carcinoma	1 (2)	0	
	Invasive micropapillary carcinoma	2 (4.1)	0	
	Lobular carcinoma	1 (2)	0	
	Medullary BC	0	1 (2)	
**Receptors status,** ***n*** **(%)**	ER positive	32 (65.3)	37 (74)	0.27
	PR positive	30 (61.2)	31 (62)	0.32
	Her2 positive	18 (36.7)	20 (40)	0.33
**Pre-CTH tumor size (cm), mean ± SD**		3.3 ± 0.6	3.2 ± 0.57	0.39
**Post-CTH tumor size (cm), mean ± SD**		1.6 ± 0.6	1.5 ± 0.58	0.13
**Clinical nodes,** ***n*** **(%)**	1	17 (34.7)	31 (62)	0.023
	2	15 (30.6)	10 (20)	
	3	17 (34.7)	9 (18)	
**TNM stage,** ***n*** **(%)**	I	3 (6.1)	3 (6)	0.15
	II	22 (44.9)	28 (56)	
	III	29 (59.1)	19 (38)	
**Type of CTH,** ***n*** **(%)**	AC + TH	6 (12.3)	13 (26)	0.17
	AC + T	43 (87.7)	37 (74)	
**No. of cycles**	4 cycles	1 (2)	1 (2)	0.34
	6 cycles	48 (98)	47 (94)	
	8 cycles	0	2 (4)	
**Radiological response**	CR	5 (10.2)	5 (10)	0.72
	PR	38 (77.6)	39 (78)	
	SD	4 (8.2)	2 (4)	
	DP	2 (4.1)	4 (8)	

*AC* Adriamycin, cyclophosphamide, *HF* Harmonic Focus, *CE* conventional electrocautery, *DM* diabetes mellitus, *HTN* hypertension, *DCIS* ductal carcinoma in situ, *ER* estrogen, *PR* progesterone receptor, *T* Taxol, *TH* trastuzumab, herceptin. Radiological response: *CR* complete response, *PR* partial response, *SD* stable disease, *DP* disease progression

In terms of intraoperative characteristics, patients in the Harmonic Focus group had significantly shorter operative times than the monopolar electrocautery group (101.3 ± 27.3 vs. 139.3 ± 31.9 min, respectively; *p* < 0.001). Besides, blood loss was significantly lower in the Harmonic Focus group (117.1 ± 35.6 vs. 187 ± 49.8 mL, respectively; *p* < 0.001). No significant differences were detected in other intraoperative characteristics (Table 2).

**Table 2:** Intraoperative data of the included patients

Variables	HF group (***n*** = 49)	CE group (***n*** = 50)	***p***
**Duration of operation (min), mean ± SD**	101.32 ± 27.3	139.3 ± 31.9	0.001
**Blood loss (mL), mean ± SD**	117.14 ± 35.6	187 ± 49.8	0.001
**No. total lymph nodes, mean ± SD**	18.24 ± 4.7	17.46 ± 5.9	0.46
**No. positive lymph nodes, mean ± SD**	3.2 ± 4.4	2.8 ± 4.7	0.69
**pTNM,** ***n*** **(%)**			
**I**	29 (59.2)	39 (78)	0.168
**I**	6 (12.2)	5 (10)	
**III**	14 (28.6)	6 (12)	

Postoperatively, patients in the Harmonic Focus group had a significantly lower volume of chest wall drain (86.8 ± 20.7 vs. 147.5 ± 35.4 in monopolar electrocautery; *p* < 0.001) and shorter time until drain removal (2.6 ± 0.7 vs. 4.1 ± 0.9 in monopolar electrocautery; *p* < 0.001). Likewise, patients in the Harmonic Focus group had a significantly lower volume of axillary drain and shorter time until drain removal than monopolar electrocautery (*p* < 0.001). The frequency of pain was lower in the Harmonic Focus group 72 h after the procedure (4.1% vs. 28%; *p* = 0.007). The incidence of postoperative complications was comparable between both groups (*p* = 0.128). The incidence of postoperative complications was similar between both groups (*p* = 0.128). Two patients developed postoperative seroma in the Harmonic Focus group, and three patients developed postoperative seroma in the monopolar electrocautery group (*p* = 0.63; Table 3). The overall seroma volume was not exceeding 100 cc per time of aspiration or initially at diagnosis of seroma.

**Table 3:** Postoperative data of the included patients

Variables		HF group (***n*** = 49)	CE group (***n*** = 50)	***p***
**Volume of chest wall drain (mL), mean ± SD**		86.83 ± 20.7	147.5 ± 35.4	0.001
**Days to chest wall drain removal, mean ± SD**		2.551 ± 0.67	4.12 ± 0.96	0.001
**Volume of axillary drain (mL), mean ± SD**		160.71 ± 32.2	283.5 ± 69.7	0.001
**Days to axillary drain removal, mean ± SD**		5.61 ± 1.8	9.74 ± 2.3	0.001
**Hospital stay (days), mean ± SD**		1.16 ± 0.37	2.28 ± 0.57	0.001
**Postoperative complications (excluding seroma),** ***n*** **(%)**	Hematoma	1 (2)	1 (2)	0.128
	Numbness	2 (4.1)	1 (2)	
	Shoulder stiffness	5 (10.2)	2 (4)	
	Wound infection	0	2 (4)	
**Seroma,** ***n*** **(%)**		2 (4.1)	3 (6)	0.63
**Pain,** ***n*** **(%)**	12 h	20 (40.9)	27 (54)	0.48
	24 h	14 (28.6)	19 (38)	0.32
	48 h	2 (4.1)	14 (28)	0.007

## Discussion

Neoadjuvant CTH can potentially increase the risk of certain postoperative complications, especially infection, owing to its associated neutropenia [5]. Nonetheless, the impact of neoadjuvant CTH on postoperative complications of BCS is still controversial. Although the Harmonic scalpel is a well-established tool in various surgeries, there is a controversy regarding its superiority over conventional methods in BCS. Moreover, no previous study has examined the superiority of the Harmonic scalpel in patients receiving neoadjuvant CTH. In this comparative study, the Harmonic Focus scalpel was associated with shorter operative time and less blood loss than conventional electrocautery. Moreover, patients in the Harmonic Focus group had a significantly lower drain volume and shorter time until drain removal than the monopolar electrocautery group and less pain on the third postoperative day. In contrast, patients in the Harmonic Focus scalpel group had comparable rates of postoperative complications to patients in the monopolar electrocautery group.

Previous reports demonstrated that drain volume and duration until drain removal are positively correlated with the risk of local infectious complications [12]. This study showed that the Harmonic scalpel led to lower drain volume and shorter time until drain removal than monopolar electrocautery [13]. These findings can be explained by induced lymphostasis, excessive thermal injury of the lymphatic system, and hematoma formation after electrocoagulation. In contrast, Harmonic Focus forms a coagulum that seals lymphatics and produces minimal damage to lymphatic tissue, which, in return, reduces drainage [14]. These findings are also hypothesized to stem from the ability of the Harmonic scalpel to deal with lymphatic vessels without reopening again [15]. This was in agreement with a previous meta-analysis of 12 studies, which demonstrated lower drain volume following Harmonic scalpel than conventional electrocautery [16]. In another two reports from China and Germany, Harmonic Focus significantly reduced the drain volume and time until drain removal compared to monopolar electrocautery among women undergoing BCS [10].

Postoperative seroma, a term used to describe an accumulation of serious fluid beneath the flap or in the axially dead space, is a common complication after BC surgery, with a reported incidence of 2 to 80% according to the nature of the procedures [17]. Although seroma is not associated with a significant increase in mortality, it can trouble the postoperative course of the affected patients by increasing the risk of prolonged draining, infection, and reoperation, which, in return, can significantly delay adjuvant CTH [17]. Surgical techniques and devices are thought to impact the risk of postoperative seroma significantly. For example, electrocautery was found to be associated with the highest incidence of postoperative seroma among surgical devices for BC surgery [8]. In contrast, the Harmonic scalpel is thought to reduce seroma incidence through minimal tissue damage, proper hemostasis, and lower risk of flap necrosis compared to other techniques. However, this study demonstrated that the rate of postoperative seroma was comparable between the Harmonic scalpel and monopolar electrocautery. In concordance with our findings, Archana et al. [15] and Selvendran et al. [18] reported no significant difference between the Harmonic scalpel and monopolar electrocautery regarding the incidence of post-BC surgery seroma. Similar findings were reported by others [19, 20]. The similar rates of seroma formation in this study’s groups despite the significant difference in drain volume can be explained by the drain placement in all cases. The drain placement itself can significantly reduce the risk of seroma formation [21].

Nonetheless, current evidence shows conflicting results regarding the role of the Harmonic scalpel in reducing the incidence of seroma, as other reports demonstrated a significant reduction in seroma following Harmonic scalpel compared to monopolar electrocautery in patients undergoing BC surgery [14, 16]. Such contradictory results can be explained by wide variations in patients’ characteristics, type of surgery, surgeon’s experience, the definition of seroma, and length of follow-up among published studies. Further, a well-designed trial with multinational collaboration is warranted to investigate the impact of the Harmonic scalpel on seroma prevention after BC surgery.

Proper hemostatic control is critical intraoperatively to reduce blood loss, time of surgery, and, subsequently, postoperative morbidity and operative expenses. As mentioned previously, conventional electrocautery is limited by excessive time for tissue dissection and wide thermal damage, which, in return, can result in excessive blood loss and prolonged operative time [22]. The Harmonic scalpel works by dividing the tissues longitudinally through high-frequency ultrasonic waves, which potentially takes less time for tissue damage than conventional methods. Besides, the Harmonic scalpel produces lower temperature than electrocautery and hence less liability to excessive tissue damage and blood loss. Finally, coagulating shears lead to the development of a coagulum that effectively seals blood vessels [23]. This comparative study demonstrated that the Harmonic scalpel had the advantage of less operative time and blood loss than monopolar electrocautery in BCS with lymphadenectomy. These findings are in line with recent systematic reviews indicating less blood loss following Harmonic scalpel than conventional methods [14, 16]; however, no previous studies have assessed the Harmonic scalpel in neoadjuvant CTH.

Older age, large tumor size, advanced tumor stage, and history of anticoagulants or tamoxifen are common patient-related risk factors for postoperative seroma [24]. Her-2-positive status was an independent predictor of seroma development in this cohort, whereas patients with seroma were more likely to have advanced TNM stages and shorter days to chest drain removal. These findings aligned with previous reports indicating significant associations between hormonal status and the risk of postoperative seroma.

## Conclusion

This study confirmed the superiority of Harmonic Focus compared to monopolar electrocautery in many intraoperative and postoperative parameters, such as operative time, amount of blood loss, drain volume, and length of drain placement among patients receiving neoadjuvant CTH before BCS. In contrast, this study found no significant difference between Harmonic Focus and monopolar electrocautery regarding the incidence of postoperative seroma and other complications. Nonetheless, Harmonic Focus is a feasible and safe technique, and it should be favored over conventional techniques in well-equipped centers. Further, a well-designed trial with multinational collaboration is warranted to investigate the impact of the Harmonic scalpel on seroma prevention among patients receiving neoadjuvant CTH before BCS.

## Data Availability

The datasets used and/or analyzed during the current study are available from the corresponding author on reasonable request. All data generated or analyzed during this study are included in this published article [and its supplementary information files].

## Abbreviations

BCS: Breast conservative surgery
CTH: Chemotherapy
AC: Adriamycin, cyclophosphamide
BMI: Body mass index
OR: Odds ratio
CR: Complete response
DP: Disease progression
SD: Stable disease
PR: Partial response
TH: Taxol, herceptin
